# Who CaREs - We do: Development of a community and researcher engagement program in translational medicine

**DOI:** 10.1186/s40900-026-00843-2

**Published:** 2026-02-04

**Authors:** Evangelia Bishop, Gabriella Tikellis, Catherine Carmichael, Narelle S. Cox, Tiffany Rushen, Anna Steiner, Ken Young, Jurie Tashkandi, Pia Campagna, Arwel W. Jones, Karen Alt

**Affiliations:** 1 Community and Researcher Engagement Program, School of Translational Medicine, Monash University, Level 6 Alfred Centre, 99 Commercial Road, Melbourne, VIC 3004 Australia; 2 Respiratory Research@Alfred, School of Translational Medicine, Monash University, Melbourne, Australia; 3 Centre for Cancer Research, Hudson Institute of Medical Research, Melbourne, Australia; 4 Department of Neuroscience, School of Translational Medicine, Monash University, Melbourne, Australia; 5 South East Public Health Unit, Monash Health, Melbourne, Australia; 6 Australian Centre for Blood Diseases, School of Translational Medicine, Monash University, Melbourne, Australia

## Abstract

**Background:**

There are increasing calls from research funders for the inclusion of community members at all stages of the research cycle. However, meaningful community engagement in translational research may be challenging to achieve, establish, and maintain.

**Main text:**

The Community and Researcher Engagement (CaRE) program for translational research was developed at an Australian University in response to this need. In this manuscript, we provide an overview of the CaRE program including its governance, structure and underlying principles. In addition, we detail the community and researcher partnerships (pairing service) which have been the core activity of the program since its launch in 2021. We further report on the characteristics of people who have engaged with the program, as well as their experience and satisfaction over a three-year period.

**Conclusion:**

The development of the CaRE program provides an important contribution to the evolving landscape of community engagement in translational research.

**Supplementary information:**

The online version contains supplementary material available at 10.1186/s40900-026-00843-2.

## Abstract (fr)

Community members - especially those with a lived experience of medical conditions - have the potential to make an important and meaningful contribution to all stages of research projects. This ranges from identifying the most important research questions, to determining the most effective ways to communicate research results to the broader public. However, ensuring that people with such experiences are involved in a purposeful way may be challenging to initiate and maintain. Our Australian University undertakes a broad range of research from laboratory research to clinical trials. We have developed the Community and Researcher Engagement (CaRE) program that involves the pairing of a community member with lived experience of a medical condition with a researcher studying that specific condition. The pairing provides an opportunity for the community member to work with the researcher to produce research that will be relevant and important. This article provides a summary of why the program was started and how it operates to support mutually beneficial and meaningful connections between members of the community and researchers. It also provides an insight into the characteristics of the people involved in the program during the first three years as well as their overall experience and satisfaction.

## Background

As much as 85% of health research may not yield tangible benefits for healthcare [1]. This lack of impact partly stems from insufficient engagement with end users - community members - during the research process [1, 2]. Historically, research directives have taken a ‘top-down’ approach whereby the medical profession, researchers, academics (and funding bodies) lead the conceptualisation and conduct of research with very little or no input from the community. The shift from a top-down to a bottom-up community-driven approach has been accelerating, resulting from an ongoing call to action by researchers, policy makers, community organisations, patient support networks, academic journals and funding bodies [3].

The increasing involvement of community members in research is proposed to lead to improved quality, validity, importance and relevance of research to end-users as well as to the general community [4, 5]. Countries including the UK, USA and Canada have led the way with community engagement in research; while an awareness of the importance and purpose of including community members with lived experience of a medical condition (commonly referred to as consumers) is increasing in Australia [6]. Community engagement provides those living with a medical condition and their carers/family members a mechanism for sharing their unique experiences, views and perspectives about what is important in terms of research focus and direction [7, 8]. In Australia, key policy directives for engaging the community in research policy has come from major Federal government supported funders (e.g. National Health and Medical Research Council (NHMRC), Medical Research Future Fund (MRFF)) who have developed guidance in conjunction with advocacy groups (e.g. Consumers Health Forum). Despite these directives there is often a ‘know-do’ gap between such rhetoric and the reality of implementing community engagement in research in a meaningful way [9].

Engagement in research is facilitated through the establishment of collaborative opportunities and partnerships between community members and researchers [10]. Meaningful community engagement in research can be challenging to establish and maintain at an individual researcher level, leading to calls for more integrated approaches that include targeted efforts at an organisational and system level [11]. For example, variations in the level of external or institutional funding and availability of support from facilitators or community coordinators lead to a wide array of experiences and outcomes, including resulting in barriers for community enagagment [11–14]. It has previously been stated that Australia have lacked the structures and mechanisms for engaging the community in health and medical research that have been found to be useful in other countries [15]. Scoping and systematic reviews have identified 53 to 65 frameworks that are publicly available internationally to support effective community involvement in health-related research, but it has been proposed that providing a range of resources that can be adapted and combined in local co-designed activities may be more useful than a single, one-size-fits-all framework [16, 17]. The likelihood of community engagement is also known to vary by scientific area and research stage (e.g. perceived benefits less intuitive to laboratory-based research [18]), presenting challenges for organisations with researchers at different points in the research pipeline [2, 11]. To this end, this manuscript reports on the establishment and development of the Community and Researcher Engagement (CaRE) program within the School of Translational Medicine at an Australian university, detailing the experiences and challenges encountered to date.

## Methods

### Setting and launch

The Community and Researcher Engagement program (CaRE) was launched in February 2021 by authors Catherine Carmichael and Karen Alt to support laboratory-based research within the Australian Centre for Blood Diseases, Monash University in Melbourne, Australia. The Australian Centre for Blood Diseases is a research centre within the School of Translational Medicine at Monash University. Other researchers from this centre were asked to join the program as a founding committee alongside two community members who were known to the researchers with a lived experience of blood cancers. Awareness of the program spread quickly across researchers of other departments and centres within the School of Translational Medicine. Due to the growing demand from researchers and potential for community engagement across foundational and clinical research at the Alfred Health and Medical Research Precinct (co-location of Monash University and one of Australia’s busiest hospitals – Alfred Hospital - in Melbourne), the program was expanded in September 2021, and offered to the wider School of Translational Medicine. The School of Translational Medicine is part of Monash University’s Sub-Faculty of Translational Medicine and Public Health and a core hub for translational research and medicine, covering a breadth of disciplines, being home to eleven departments and five centres/institutes including the aforementioned Australian Centre for Blood Diseases. Following expansion of the CaRE program, a community engagement coordinator was appointed and a larger program committee was sought, which was representative of the range of disciplines and stage of research according to translational pipeline. Researchers were identified by self-invitation to the committee or direct invitations from the chairs to staff known to have experience in community engagement. Emails were sent School-wide to specifically seek expressions of interest from early-to-mid career researchers to join the committee. Community members were identified from, those who had expressed an interest to join the program by a community engagement coordinator or, recommendations by local foundations.

### CaRE overview, governance and structure

**Fig. 1:**
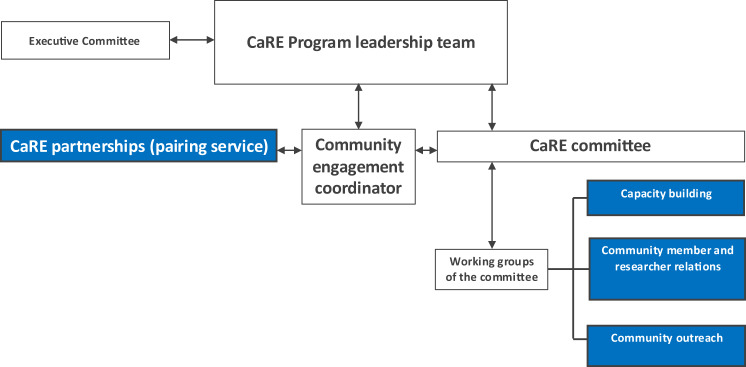
Governance structure of the CaRE program

The governance structure of CaRE is outlined in Fig. 1. CaRE is supported by a 0.4 Full-Time Equivalent community engagement coordinator and steered by a committee comprising community representatives and researchers in a mandatory minimum ratio of one community member to three School of Translational Medicine staff. The researchers represent seven departments and research institutes within the School of Translational Medicine. The committee operates on a voluntary basis and is governed and guided by Monash University policies and procedures, and a locally developed and ratified Terms of Reference. The decisions of the committee, led by a Chair and Deputy Chair, are operationalised by three working groups: capacity building; community member and researcher relations; and community outreach (Table 1). The activities of the community member and researcher relations and community outreach working groups often involves connecting with peak bodies (advocacy groups or non-government organisations representing interest of a specific community), community organisations and foundations.

**Table 1:** Working groups of the CaRE committee

**Community member and researcher relations:** *working towards creating and maintaining successful research partnerships*
• Recruit community members/researchers to participate in collaborative research opportunities. • Create connections with relevant peak bodies, community organisations, and foundations to assist with recruitment of community members for research partnerships and research collaborations. • Create promotional material to recruit community members for research partnerships. • Develop orientation and other educational resources for community members and researchers new to community involvement in research and CaRE. • Collate and produce content for community member email updates. • Collate and produce content for internal researcher news channels. • Ensure CaRE systems to coordinate community and researcher engagement and the record keeping database are in place, efficient and meet legislative, policy and procedural standards.
**Capacity building:** *working towards improving the profile and understanding of the value of the CaRE program within the School of Translational Medicine*
• Undertake projects that educate/inform the School of Translational Medicine researchers and researcher-clinicians about: - What is genuine and authentic community member engagement, as opposed to tokenistic engagement. - The purpose of community member engagement in medical research. - Methods of community engagement in medical research. - Solutions to challenges of community member engagement and achieving successful research partnerships. • Collate and produce content for social media and internal researcher news channels.
**Community Outreach:** *working towards building the profile of CaRE in the community, and sharing research with the community*
• Create connections with relevant community organisations and foundations to promote the CaRE program and highlight research being conducted within the School. • Collate and produce content for social media, internal researcher news channels that highlight the CaRE program and school of translational medicine research. • Maintenance of CaRE program website. • Special event ideas generation and project management. • Seminars/webinars to raise awareness of research or specific diseases or conditions. • Social media impact campaigns. • Offering student internship placements.

**Fig. 2:**
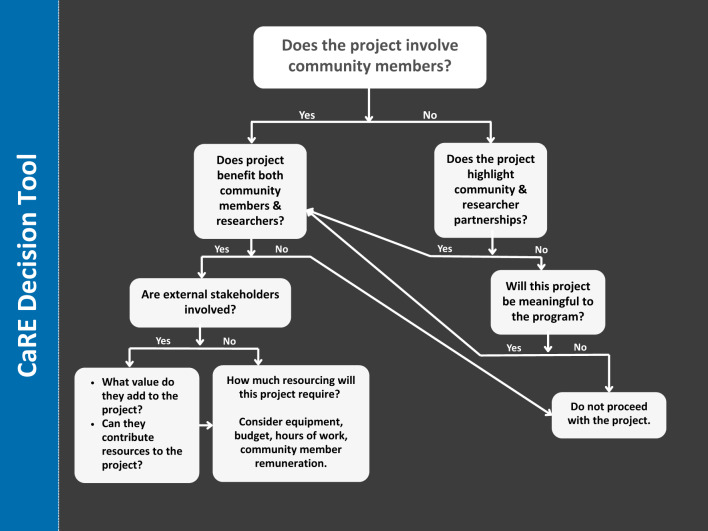
A decision tool to guide the support of activities undertaken by the CaRE committee

The CaRE committee typically meets for 1 hour, on a six-weekly basis to discuss the strategic direction of CaRE and activities of the working groups. The committee endeavours to reach decisions by consensus, however a community representative of the committee must always inform any decisions. If a community member is not present, any decisions are deferred. In the absence of consensus, a resolution of the group must be supported by a simple majority of those group members present and voting. Discussions are supported through a decision tool (Fig. 2). In addition to the working groups, a weekly operational meeting between the Chair, Deputy Chair and community engagement coordinator is held, and an annual strategic planning session is completed in conjunction with School research manager to ensure continuous progress. Every two months, the Chair or Deputy Chair reports on program activities at a School Executive committee meeting, which includes leadership of the school and its departments and research institutes.

Since CaRE’s inception, all operational structures, frameworks, policies, guidelines, and tools have been collaboratively developed between the researchers and community members of the committee. This collaborative process between community members and researchers has sought ad-hoc support from academic administrative staff and the Monash University legal team on as needs basis to ensure comprehensive coverage of all relevant aspects (e.g. involvement of the legal team to execute confidentiality agreements). This approach has supported the overall goal of CaRE, which is to facilitate mutually beneficial and meaningful connections between researchers and members of the community who have lived experience of the medical condition/s studied by the School of Translational Medicine.

**Fig. 3:**
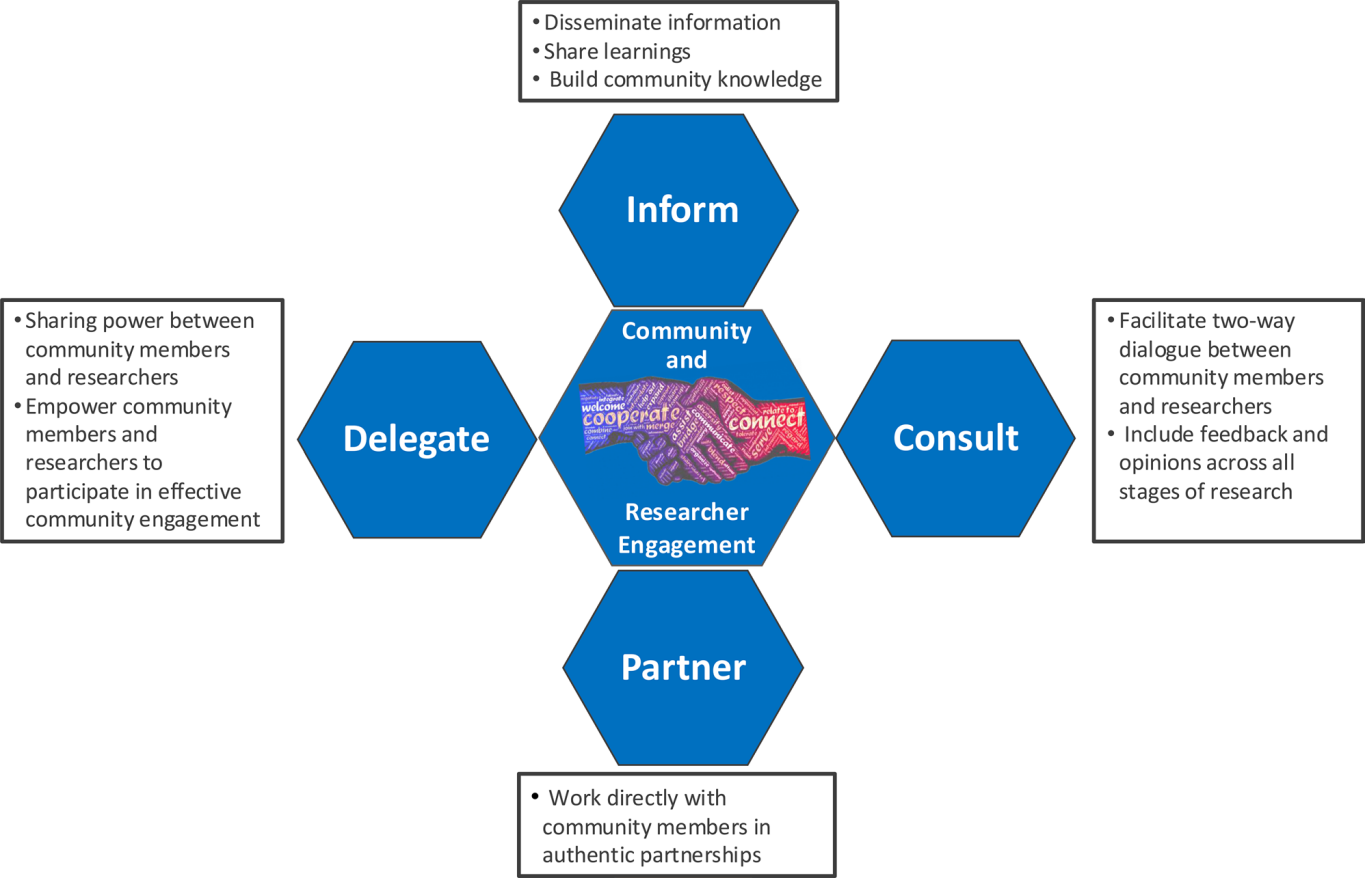
CaRE engagement approaches

CaRE is underpinned by a person-centred philosophy, prioritising free and open dialogue between the community and researchers as its central principle. CaRE seeks to deliberately remove the power dynamics often associated with hierarchy in researcher-community or clinician-patient relationships, focusing instead on collaboration and shared understanding. CaRE is motivated by a commitment to inclusive research that is meaningful to and usable by the community, but which also meets the expectations of funders who are increasingly sharing this commitment. Therefore, CaRE adopted four engagement approaches [19]: inform, consult, partner, and power delegation (Fig. 3). These approaches guide CaRE towards effective community engagement for improved health and medical outcomes, and acknowledges that differing levels of participation are legitimate depending on the people, goals, timeframes, resources and levels of concern in the decision(s) to be made.

### Community and researcher partnerships (pairing service)

At the core of CaRE is a pairing service whereby members of the community are matched with a researcher or research team. Each partnership begins with an expression of interest from a community member or researcher to the CaRE email address or online form (available in English only) on the program website. The expressions of interest form for community members asks for name, gender, location, lived experience (patient, carer, other), research areas they would like to assist (list of medical conditions), and contact details. The expressions of interest form for researchers asks for name, position at the School (student, researcher, group leader, other), department, research areas they would like assistance from the community (list of medical conditions) and contact details. The community engagement coordinator is the first line of contact for both researchers and community members and is integral in the formation and ongoing monitoring of partnerships. CaRE supports researchers in identifying relevant community members through local and social media advertising when a suitable match cannot be found within the existing community member database. Conversely, community members who are not immediately paired with a research project can remain on the CaRE database for future opportunities or choose to contribute to the program in other ways, such as providing feedback on program development.

Establishment of a formal partnership between a community member and researcher first involves an invitation to complete a confidentiality agreement. This process supports researchers and community partners to confirm suitability and to set expectations, prior experience, time and frequency of commitment, and remuneration. Remuneration of community members, whilst acknowledged as best practice, is requested but not currently mandated in any partnership. In practice, community member remuneration is more achievable for activities or projects supported by external funders. These partnerships represent a key driver for the CaRE program achieving authentic community engagement. The intention for these established partnerships is to go beyond superficial exchanges or tokenistic engagement (e.g., “last minute” or “tick box” need), by fostering open communication, mutual benefit, and ultimately improved disease and/or illness outcomes. Written and digital (podcast) guides are provided to researchers and community members to help them start, contribute to, and maintain effective community engagement partnerships. Regular oversight of the resources by committee community members has been integral to ensuring that CaRE has been developed and continues to develop in accordance with effective and respectful community involvement.

CaRE promotes community member-researcher collaboration throughout all stages of the research cycle. The nature and scope of community members’ involvement will vary between research projects depending on the interest and availability of the community member(s) and needs of the research team. As a means of supporting initial engagement and partnership building, CaRE suggests researchers invite community members to meet the research team via lab tours, onsite visits (subject to community members’ accessibility and health status), laboratory meetings and plain language presentations of the research area and projects. Other collaborative stages include project initiation and significance definition, planning and consultation phases, grant application and outcome processes, clinical trial or project management, and dissemination of research findings and project outcomes.

CaRE has implemented regular monitoring and conducted an internal review of its first three years to assess impact, review partnerships, and identify areas for improvement. Characteristics of community members and researchers who have expressed an interest in joining CaRE and feedback from those involved in the program and its partnerships have been collected to inform ongoing development. Expressions of interest form data have been collected and managed using REDCap (Research Electronic Data Capture) electronic data capture tool hosted and managed by Helix (Monash University) [20, 21]. REDCap is a secure, web-based software platform designed to support data capture, providing 1) an intuitive interface for validated data capture; 2) audit trails for tracking data manipulation and export procedures; 3) automated export procedures for seamless data downloads to common statistical packages; and 4) procedures for data integration and interoperability with external sources. Annual surveys of the CaRE the program have also been hosted in REDCap. Each year community members and researchers were sent links to the surveys via email. Community members and researchers were asked ‘To what extent would you say you are satisfied with your experience of the CaRE program?’, with options of very satisfied, satisfied, unsure or not satisfied. Researchers were additionally asked ‘To what extent, if any, has being part of the CaRE Program changed your perception of consumer engagement’, with options of significantly, somewhat, or not at all. Anonymous, open-ended responses from community members and researchers in annual surveys (since 2021) have also been sought on the following areas: overall experience with the CaRE program (community member and researcher); main outcomes or impacts of being in a partnership (community member and researcher); any recommendations of the program to others (community member and researcher), contact with a formal coordinator (community member and researcher); contributions to research (community member); changes in perception of working with the community (researchers); and resources or support to enhance engagement with community members (researchers). Free text responses were categorised at a high level and tabulated.

These data are presented in the subsequent sections and offer valuable insights into the development of CaRE. Two community committee members who were involved in CaRE from its inception were also invited to a recorded interview to share their experience of being involved in the program. They were asked to reflect on how CaRE distinguishes itself from other community engagement programs. Verbatim open ended survey responses and interview extracts have been tabulated and grouped into categories.

## Results

### Recruitment of community members into CaRE

From 2021 to 2023 (inclusive), a total of 62 community members expressed an interest in joining CaRE to contribute to medical research (Table 2). Overall, community members were individuals with lived experience of a medical condition, however interest was also received from carers of people living with a medical condition. Most of the community members identified as female and resided in Australian urban and regional localities. There was a range of medical conditions represented by people with lived experience including encephalitis, myeloproliferative neoplasms, epilepsy, and dementia. Across the three years, nearly half of all community members were paired with a researcher or research team, with most of these engagements ongoing at the time of writing (Table 2).

**Table 2:** Characteristics of community members engaged in CaRE in 2021–2023 inclusive

Characteristic	2021 N = 44 n (%)	2022 N = 10 n (%)	2023 N = 8 n(%)	TOTAL N = 62 n (%)
**Community member role**				
Person with lived experience	24 (54)	8 (80)	5 (63)	37 (60)
Carer	18 (41)	2 (20)	3 (37)	23 (37)
Lived experience and carer	2 (5)	0 (0)	0 (0)	2 (3)
**Specific lived experience condition/illness**				
Acute myeloid leukemia	0 (0)	3 (30)	0 (0)	3 (5)
Encephalitis (autoimmune, GFAP)	9 (20)	1 (10)	0 (0)	10 (16)
Cancer (breast, colorectal, lung, neuroendocrine, unspecified)	0 (0)	4 (40)	4 (50)	8 (13)
Cardiovascular disease	2 (5)	0 (0)	0 (0)	2 (3)
Chronic pain	0 (0)	0 (0)	1 (13)	1 (2)
Dementia (includes Lewy body, Alzheimer’s disease)	12 (27)	0 (0)	0 (0)	6 (11)
Diabetes	1 (2)	0 (0)	1 (13)	2 (3)
Epilepsy (includes Traumatic Brain Injury)	6 (14)	1 (10)	1 (13)	8 (13)
Inclusion body myositis	1 (1)	0 (0)	0 (0)	1 (2)
Multiple sclerosis	0 (0)	1 (10)	0 (0)	1 (2)
Myeloproliferative neoplasms	0 (0)	9 (90)	0 (0)	9 (15)
Non-Hodgkin’s Lymphoma	0 (0)	2 (20)	1 (13)	3 (5)
SCA36 -Motor neuron disease	1 (2)	1 (10)	0 (0)	1 (2)
Stroke	1 (2)	1 (10)	0 (0)	2 (3)
**Gender**				
He/Him/His	6 (14)	5 (50)	3 (38)	14 (22)
She/Her/Her	32 (72)	3 (30)	4 (50)	39 (63)
Not provided	6 (14)	2 (20)	1 (13)	9 (15)
**Geographic location**				
Urban	10 (24)	7 (70)	6 (75)	23 (37)
Regional	15 (34)	2 (20)	1 (13)	18 (29)
Not reported	19 (43)	1 (10)	1 (13)	21 (34)
**Residential state/territory in Australia**				
Australian Capital Territory	0 (0)	0 (0)	0 (0)	0(0)
New South Wales	2 (4)	1 (10)	2 (25)	5 (8)
Northern Territory	0 (0)	0 (0)	0 (0)	0 (0)
Queensland	2 (4)	2 (20)	1 (13)	5 (8)
South Australia	0 (0)	1 (10)	1 (13)	2 (3)
Tasmania	2 (4)	0 (0)	0 (0)	2 (3)
Victoria	32 (73)	6 (60)	4 (50)	42 (68)
Other – United States	3 (7)	0 (0)	0 (0)	3 (5)
Not reported	6 (14)	0 (0)	0 (0)	3 (5)
**Paired with Researcher, yes**	16 (36)	5 (50)	6 (75)	27 (43)
**Engagement ongoing, yes**	35 (80)	8 (80)	8 (100)	51 (82)
**Communication preference**				
Face to face	19 (43)	2 (20)	4 (50)	25 (40)
Phone	8 (18)	5 (50)	4 (50)	17 (27)
Video meeting	18 (41)	3 (30)	0 (0)	21 (39)
No preference	14 (32)	3 (30)	0 (0)	17 (27)

### Recruitment of researchers into CaRE

In the evaluation period, 34 researchers expressed an interest in joining the program (Table 3). Primarily these were senior researchers. Focus topics among researchers varied, with many investigating acute myeloid leukemia and myeloproliferative neoplasms, followed by epilepsy and dementia. The pairing of researchers with community members was successful in the first two years, with an overall match/pairing rate of 62%, whereas in 2023 interest was expressed from researchers in areas where the program did not have community members with the lived experience being sought.

**Table 3:** Characteristics of researchers engaged in CaRE in 2021–2023 inclusive

Characteristic	2021 N = 13 n (%)	2022 N = 12 n (%)	2023 N = 9 n (%)	TOTAL N = 34 n (%)
**Academic position**				
Group leader	8 (62)	4 (33)	4 (44)	16 (47)
Research assistant/post-doctoral fellow	5 (38)	5 (42)	4 (44)	14 (41)
Research managers or administrators	0 (0)	3 (25)	1 (12)	4 (12)
**Specific condition of research interest**				
Acute myeloid leukaemia & myeloproliferative neoplasms	2 (15)	2 (17)	0 (0)	4 (12)
Encephalitis (autoimmune)	1 (8)	0 (0)	0 (0)	1 (3)
Chronic post-surgical pain	0 (0)	1 (8)	0 (0)	1 (3)
Colorectal cancer	0 (0)	1 (8)	0 (0)	1 (3)
Dementia	2 (15)	0 (0)	1 (11)	3 (9)
Diabetes	1 (8)	1 (8)	1 (11)	3 (9)
Epilepsy	0 (0)	3 (25)	1 (11)	4 (12)
Group A streptococcus infections	0 (0)	0 (0)	1 (11)	1 (3)
Hospital acquired infections	0 (0)	0 (0)	1 (11)	1 (3)
Motor neuron disease and Amyotrophic lateral sclerosis	1 (8)	1 (8)	0 (0)	2 (6)
Multiple myeloma	1 (8)	0 (0)	0 (0)	1 (3)
Multiple sclerosis (neuroimmunology)	2 (15)	0 (0)	1 (11)	3 (9)
Obesity	0 (0)	0 (0)	1 (11)	1 (3)
Ovarian cancer	0 (0)	1 (8)	0 (0)	1 (3)
Stroke/brain injuries, early life brain insults	2 (15)	2 (17)	0 (0)	4 (12)
Respiratory diseases (asthma, bronchiectasis, COPD)	0 (0)	0 (0)	1 (11)	1 (3)
Thrombosis, heart failure	1 (8)	0 (0)	0 (0)	1 (3)
Transfer of drugs during pregnancy	0 (0)	0 (0)	1 (11)	1 (3)
**Paired with Community Member, yes**	9 (69)	11 (92)	1 (11)	21 (62)

### Community and researcher satisfaction and experiences

Pivotal to the development of the CaRE program has been the community members and the researchers involved. Their feedback and reflections have been crucial and continue to underpin the aspirations, goals, planning and activities of CaRE.

### Community member satisfaction and experiences

Between 2021 and 2023, 17 of 62 (27% response rate) community members provided their level of satisfaction in the program, with 47% (*n* = 8) saying they were ‘very satisfied’, 41% (*n* = 7) satisfied, 6% (*n* = 1) were ‘unsure’, and 6% (*n* = 1) was ‘not satisfied’. Anonymous, open-ended responses from community members via annual surveys since 2021 are reported in Table 4.

**Table 4:** Community members’ reflections on CaRE since expressing an interest in joining the program

**Community members’ descriptions of** **overall experience** **of the CaRE program**	**New understanding of the studied disease** • “I feel like there is opportunity to have my experience be recognised and that medical professionals can always do better to understand what a patient with a chronic lifelong condition is going through.” • “The CaRE program is wonderful for patients and or carers. It allows both to have not only knowledge but input into how tomorrow’s clinicians support those with the disease or affected with the disease. It allows for information to be disseminated in an understandable way for those who need it and not just read by clinicians.” **Supportive with no tokenism** • “No tokenism, real partnership.” • “Supportive and overall interested in new ideas etc that are offered.” • “Everyone involved that I have spoken with has been wonderful. Coordinator is a star!” • “The program is very rewarding, to be involved and asked as a patient to contribute is a special privilege. Also to be shown around a research facility and meet some researchers was a wonderful experience.” • “I have enjoyed CaRE it has been enormously helpful. The researchers are fantastic, informative, caring.” **Interesting and unexpected** • “Absolutely excellent! Worthwhile, rewarding, engaging, interesting, positive.” • “Very interesting and informative.” • “So far, it’s been interesting.” • “Not what I expected - this could be mutual i.e. the research team’s experience also.” **Importance of matching with researcher** • “Hopefully someone else would be better matched with the research program & would be able to provide valuable input” • “Research is very deep technical - my lived experience as a carer too far removed from/for the research team.” • “I’ve had limited contact with my researcher and haven’t been offered other opportunities to contribute.” • “This program is unique because there is NO middle man, a consumer manager who manages consumers essentially to support brand leveraging activities and basically is using consumers as a marketing tool. This program is genuine.” • “At this stage my interest relates to strokes.” **Engaging outside of partnership** • “Would like to be more active as per original contact but enjoy emails with updates keeps me informed what is happening out there in this space but any more can’t commit due to work and 24/7 caring for parent with dementia in family home Thank you for continuing to email updates.” • “email is easier for me.”
**Community members’ descriptions of** **main outcomes or impacts** **for community members, of being part of a CaRE partnership**	**New knowledge** • “Increase of knowledge.” • “Added knowledge.” • “New thoughts for clinicians on how to recognise and diagnose this disease early. New ways for clinicians to communicate to patients/carers. Greater local support for carers and patients.” • “I have improved my knowledge which has helped my advocacy for my daughter and for a number of other carers. The research is amazing that is shared with us.” **Making a difference on other people living with same disease** • “It’s a privilege to be part of a CaRE partnership, to be heard as someone living with an incurable blood cancer, to be asked about my lived experience and to know I am genuinely valued and respected in this space. This opportunity gives so much positivity and enables patients and carers to contribute during/after very challenging personal experiences, so others can benefit and live healthier, longer lives.” • “It’s a great way to learn about research and how you can inform research practice. Also therapeutic to share your story and know that you’re making a difference.”
	**Feeling involved and a sense of purpose** • “Feeling that there is a genuine partnership between researcher and consumer. When i assess grant proposals i compare this program of consumer involvement with what other researchers do in the space. It is my benchmark to evaluate pretence versus substance.” • “Happy to be involved where needed to help contribute to MPN research.” • “Feeling more involved has given me more confidence in discussing my illness with others including medical specialists and other patients. Also an understanding of the difficulties researchers face in getting funding to such important research, whatever I can do to help is the least I can do.” • “It’s good to be connected to research studying my condition and to contribute as a consumer.” • “A sense of purpose. Satisfaction from creating positive changes to research projects. Knowing that I’m helping to educate researchers about lived experience.” • “It’s always wonderful to see how your story has impacted the researcher and how they have improved their study after speaking with you. It makes the sadness of the disease ease a little because you’ve seen some good come out of it.” “I feel like there is opportunity to have my experience be recognised and that medical professionals can always do better to understand what a patient with a chronic life long condition is going through.” • “Happy to continue participating in this and similar projects.” **Impact unclear** • “Too early to say.” • “Nil – unfortunately.” • “Too early to tell.” • “little to offer in research area.” • “Minimal involvement/request/opportunity to date.” • “Not sure at this stage I hope I have been of some assistance.”
**Community members descriptions of their** **contributions** **to research**	**Informing grant proposals and research design** • “After reviewing a researcher’s grant application document, they genuinely acknowledged my input and made changes to the document prior to submission. Researchers often comment how positive the relationship has been, how much I have contributed and how much they value a consumer perspective.” • “I provided input on the lay statement for a grant proposal.” • “consumer reviewing research proposal and engaged in the project.” • “My story assisted with the study design.” • “Helping with a survey form on epilepsy side effects.” **Sharing lived experience** • “By sharing my story and the effects of the disease.” • “A patient story, my personal experience and positive feedback to the research team.” • “By being able to read research papers, and proof them in everyday language By giving our story to researchers and help them understand from a patient and carers view.” • “Providing feedback for epilepsy studies to help the general public understand what medical jargon means.” • “By linking bench work on mice with human experience of people living with cancer.” • “I haven’t seen any of the papers/studies as yet (happy to do so), but I feel at this stage I have contributed to the program through participating where I can and being paired with a researcher who knows they can contact me if they need.” • “My views have been sought and acknowledged.” **Unclear on contribution or relevance of research** • “little to offer in research area” • “Minimal involvement/request/opportunity to date.” • “Not sure at this stage I hope I have been of some assistance.” • “My lived experience as a carer too far removed from the very technical research being undertaken.” • “distance’ between research team & myself too great - they are immersed in research, my personal experiences not really useful and/or relevant to the team. We could be a mis-match.” • “Due to mental health issues I haven’t be able to engage as much as I wanted.”
**Reasons** **community members would recommend the CaRE program to other community members**	**New knowledge for all impacted by the disease** • “To acquire the knowledge required for patient centred care.” • “It’s an excellent program with benefits for everyone involved.” • “The CaRE program is wonderful for patients and or carers. It allows both to have not only knowledge but input into how tomorrow’s clinicians support those with the disease or affected with the disease. It allows for information to be disseminated in an understandable way for those who need it and not just read by clinicians.” • “Anyone impacted directly/indirectly by dementia, Alzheimer’s should learn as much about the illness as possible.” • “I would love to continue with CaRE so I can help others in the same position and also help researchers get knowledge of this disease into the community.” **Gratification from sharing lived experience** • It’s a great way to learn about research and how you can inform research practice. Also therapeutic to share your story and know that you’re making a difference. • “It is gratifying to contribute to research on your condition.” **Value for community members** • “Interesting information on current research.” • “I think it is good for consumers to be involved in research.” • “The research and knowledge has been invaluable and the kindness of all involved.”

CaRE has demonstrated a positive impact on community members, resulting in increased knowledge of research processes and a deeper understanding of their medical conditions. By fostering supportive and collaborative partnerships, CaRE has empowered community members to actively contribute their lived experiences to research, leading to a heightened sense of purpose and engagement, and feelings that they can make a difference on the lives of other living with similar conditions. Some community members, however, reported that it was too early to tell whether CaRE had any impact on them, felt that there had been none to date or that they were unable to contribute because of the research they were matched with. Overall, CaRE established itself as a valuable and gratifying opportunity for the community to share their story to inform funding proposals and research design.

### Researchers’ experiences

Between 2021 and 2023, 15 of 34 (44% response rate) researchers reported on their level of satisfaction in the program’, with 53% (*n* = 8) saying they were ‘very satisfied’, 27% (*n* = 4) satisfied, and 20% (*n* = 3) were ‘unsure’. Researchers (16 of 34, 47% response rate) also reported on the extent that the CaRE Program had changed their perception of consumer engagement, with 50% (*n* = 8) saying ‘significantly, 38% (*n* = 6) ‘somewhat’, and 13% (*n* = 2) were ‘not at all’. Anonymous, open-ended responses from researchers via annual surveys since 2021 are reported in Table 5.

**Table 5:** Researchers’ reflections on CaRE since expressing an interest in joining the program

**Researchers’ descriptions of** **overall experience** **of the CaRE program**	**New insight on the value of lived experience** • “CaRE has given me greater insight into the patient/consumer perspective and journey.” • “Broaden your perspective as a researcher, you never know where this type of thing will need or develop into. For consumers, perhaps involvement is a way to feel engaged with and have some resemblance of control over their condition.” • “More researchers should get the interesting and important perspectives of the educated lay audience” • “I see it is possible and having a connection to a consumer makes my work feel more valuable.” • “I did not think the consumers would want to read Grant proposals but some of them.” **Connection with the community** • “Being part of the program has given me confidence to work with consumers and communicate better with members of the public/those affected by the conditions that I study. Their insights have also been really valuable for reminding my team why we do what we do, and that the patient is the ultimate end point.” • “It is a great program, the support is amazing, and it makes it so easy for a researcher to connect with a consumer and develop a beneficial partnership.” • “Great support in establishing connections.” • “Providing a structure and framework for forming partnerships with consumers.” • “Helped link us with a person with lived experience of a condition to inform our study.” • “Provides me with an avenue to directly reach someone affected by the research that I do (as a non-clinician, this is sometimes hard to do organically).” • “It is very important as the CaRE program already has consumers looking for projects to be associated with.” **Understanding of the program** • “A resource providing structure and guidance to assist researchers to develop and maintain mutually-beneficial relationships with a member(s) of the public who are affected by the disease they study.” • “A program that aims to connect consumers with researchers and help facilitate that connection and partnership.” • “A committee to facilitate connections between researchers and consumers.” • “The committee facilitates the interaction between scientists and consumers and organises events to grow those relationships.” • “A way of communicating with the end users of our research” • “Consumer engagement.” • “Very worthwhile and it provided a necessary perspective of the value of research projects from the eyes of a consumer.” **Needs are met** • “I have what I need from the program.” • “It provides an avenue on my behalf to match my needs with a consumer.” **Limited engagement** • “In some ways it has helped, but in others it has been neutral. Much of my engagement has happened independent of CaRE through introductions via clinicians and other methods of engagement.” • “OK so far. Consumer is from the country so have not yet had a face to face.” • “No. My consumer has a rare form the disease, and so whilst broadly interested, our research is not directly relevant to them. We need to identify consumers to whom our area of research is more directly relevant to obtain better input and engagement.”
**Researchers’ descriptions of** **main outcomes or impacts** **of being part of a CaRE partnership**	**Contact with community member** • “The program has been very useful to get in contact with a consumer and engage with them.” **Increased understanding of the studied disease** • “So far, increased understanding about the personal toll of disease on an individual, and their self-awareness of where the research and knowledge is lacking (and what they would like to see done).” • “Better connection with consumers, greater understanding of the patient experience - my lab group members have also gained great insight into the disease we work on.” • “Provided insight into the resources within the community for those living with the disease.” • “more valuable.” **Impact unclear** • “Nothing as yet.” • “Nothing as yet as we only just commenced.” • “I was matched with a consumer and after a slow start, we now have a good interaction.” **Other** • “Consumer engagement. Awareness of basic research to the community.”
**Researchers’ descriptions of any changes in perception in working with the community**	**Pre-existing perceptions of the community engagement** • I have always believed consumer engagement was important but had previously struggled to find willing parties to engage with.” • “I think I had a good idea of what a scientist-consumer relationship could look like so the actual interaction was mainly as expected.” • “A large proportion of my consumer engagement has happened outside of the CaRE program.” • “I already recognise the importance of consumer engagement." **Awareness and confidence to involve the community** • “Participation in the program has given me greater insight into the patient/consumer perspective and journey, as well as the confidence to connect with them as a scientist and human - my communication with them doesn’t need to be perfect, and we can meet in the middle to come to mutual understandings.” • “Being part of the program has given me confidence to work with consumers and communicate better with members of the public/those affected by the conditions that I study. Their insights have also been really valuable for reminding my team why we do what we do, and that the patient is the ultimate end point.” • “Consumers can provide support post study design rather than just in the development stage.” • “Made me aware that consumer engagement doesn’t need to be on a huge scale (e.g. connection with or recognition by community groups), but can work well at an individual partnership level to being insights to both parties.” **Enthusiasm and understanding of community engagement** • “Having met with the two consumers interested in my field of research, I am more enthusiastic with this program.” • “Consumer involvement is important to the research.” • “I have really learnt how valuable connecting with a consumer is and how much I can learn about the disease from the consumer. Before engaging with the consumer I had limited appreciation of the benefits of involving a consumer/s in my research.” • “I better understand the patient’s perspective.” • “It was really good to have the consumer’s input on our study, and we altered some parts of our study based on their input.” • “It is refreshing to hear the comments of the consumers and their appreciation of the research being undertaken. I also appreciate their point of view and also how the consumers themselves are well connected within their communities and can help to spread the word of the research being undertaken by our laboratory.”
**Researchers descriptions on support from community engagement coordinator**	**Overcoming challenges with partnership** • “They were very understanding and assisted to resolve the issue in a timely and collegial manner.” • “I stopped receiving any correspondence from my consumer. I was very happy with the strategy and response received.” • “When a partnership was no longer needed due to a change in research direction (lack of funding), the coordinator was crucial for negotiating an amiable cessation of the partnership. Great response and assistance provided, very understanding.” **Point of resource and advice** • “Someone needs to be a broker or intermediary who is not directly involved for support and material provision.” • “The coordinator keeps the engagement with consumers active.” • “Imperative for initiating and supporting partnerships.” • “Coordinator was really helpful in connecting us and we appreciate her overseeing our first meeting together.” • “Always good to have somebody you can ask questions when something comes up.” • “Very useful - absolutely essential to the smooth running of the program, and reflecting the value placed on consumer engagement.” • “Initial meetings with coordinator were very helpful and made the process extremely easy. Information provided about expectations for the partnership were great. Coordinator is always available for questions and to help guide the partnership.” • “I was matched with a consumer and got useful advice on how to get going and grow the relationship.” **Unclear** • “Whilst support was helpful it was not as extensive as needed.”
**Researchers’ recommendations for enhancing community engagement**	**Check ins and reminders** • “Prompts over time to remind me to engage, and examples on how to do this, would be helpful. e.g. reminders to send consumers grant proposal drafts, manuscripts, to reach out and check how they’re doing.” • “Check in, guidance on how to build the relationship from the early foundations, would always be welcome (while appreciating that every partnership is different).” • “Check in over the first few months to assist in getting the relationship up and running - that is the hardest bit.” **Increase connections with the community** • “Open the program to the general public (not just patients/families with X disease.” • “The biggest issue has been finding connections.” **More structured support and advice** • “More along the lines of what we should be doing and things we might need to do to enhance engagement.” • “Mentorship, check in by the engagement co-ordinator.” • “Examples of ways to engage, reach out, start and then keep the engagements going would be great. What has worked well for others in the past for these types of relationships.” • “Mentorship.” • “More facilitation of engagement.” • “I feel that it needs greater clarification on the aims and what is provided through the program. I’m not actually sure it is a program, rather it is more a service to connect a researcher and a consumer.” **Need for funding** • “Financial resources to support consumer transport for meetings. We don’t have much by way of spare money to pay for taxi’s for highly disabled consumers.” • “Whilst there have been some seminars arranged discussing consumer engagement, there’s not much support in terms of helping us engage our consumers more regularly (funding required).”
**Reasons** **researchers’ would recommend the CaRE program to others**	**Importance of the community engagement coordinator** • “Professional and friendly manner of the committee and administrative support team.” • “The coordinator keeps the engagement with consumers active.” • “Having a professional expert match me with a consumer.” • “The coordinator has been absolutely an asset, wealth of knowledge and support and support and invaluable.” **Learning and reinforcement** • “The ‘work in progress’ feel of the program, and willingness to learn and grow as we go along. The up-front expectations and signatures required to ensure confidentiality and ensure some level of commitment from both parties.” • “The community member opinion is very important and insightful. The program reminds us why we do these projects in the first place.” **Other** • “Unsure.” • “Helpful.” • “Hard to say given my research is only peripherally relevant to my consumer.” • “Project and grant feedback.”

Impact of CaRE has been largely positive, with researchers recognising its value in facilitating connections with community members and gaining new insight on the contribution of lived experience to research. Researchers have expressed increased confidence in working with the community and awareness of how this can be achieved. Some of the researchers, like the community members, felt that the impact of the program remains unclear and there had been limited engagement. This included researchers who already understood the importance of community engagement prior to joining CaRE and much of their work has happened outside of the program. Many researchers reported that the role of the dedicated community engagement coordinator was invaluable in facilitating partnerships or addressing challenges. Researchers did, however, make recommendations for areas of improvement in the program. This included mentorship, check-ins and reminders, as well as funding to engage with the community more frequently or to reimburse out of pocket expenses.

### Community members’ reflections on the CaRE committee

Reflections from two community committee members who were involved in CaRE from its inception are provided in Table 6.

**Table 6:** Reflections from two community members on the CaRE committee

**Sharing of power**	*“The name says so much, and that’s not, I don’t want to be corny, but it does. And actually, not just the acronym, but the words say what they do. This clearly is, you know, engagement between two sides. Might sound minor, but it’s important.”* “*I think the critical factor was acceptance, right? From the beginning, the patients were partners. That was a debate that didn’t have to be run. It was given. And I think that that makes it. That’s the first and that’s often the hardest thing for researchers to let go of. And I think that to me that was the difference than some of the other.”*
**Transparency**	*“Some of the other programs was still fairly rule bound. One of the things I liked about the CaRE team and the CaRE Committee was that there wasn’t rules there. It was pretty blue sky and rather than ruling things out or in.”* *“It was a real sense of what is it we’re trying to do? How are we going to get there? And so there was that discussion that was open and honest. And if it can retain that, I think that’s where it’s success*.”
**Program commitment**	*“I think, the institutional support, having employed (the coordinator) and you know, the committee, and you see how long everyone’s involved on a voluntary basis is really important as well. And I would say the core group, and I don’t mean core as in the people who have hung around, but the heart of CaRE has kept pumping strongly for several years already, and the turnover is minimal, you know. I think once somebody says they’re on the committee, they’re there.”*
**Program structure**	*“I think the forward looking, you know, setting up a structure and everything is what sets (CaRE) apart from other places that sometimes you could sign up, you know, they send out an SOS for people, and you sign up, and then you don’t hear back for months. There’s no question of the commitment and, you know, focus that’s in this whole team, definitely.”*
**Genuine community engagement**	*“There was a genuine exploration, and ideas were finally turned down or accepted after a process of debate, and after a process of reflection, and I felt included in that process. I didn’t feel decisions were made elsewhere and then presented. This is what we’re going to do, or this is all we’re allowed to do. I’ve been in some. I’ve been involved in some things as a consumer where I feel the decisions are already preordained and pretty discussed. And there’s and it’s just this is what we think. This is a good idea. What do you think. And then we’re going to go on with that real idea. Whereas in the original working group. It was a real, genuine exploration."*
**Productivity**	*“And the subcommittees that we’ve had have been productive, and there’s commitment there as well, and I haven’t seen that maybe as strongly everywhere else, or unfortunately, they haven’t formed subcommittees, and you have one big committee trying to run 11 different things which hasn’t gone as efficiently.”*

## Discussion

CaRE provides a unique university-based community engagement program, which through formal community and researcher partnerships alongside other community engagement methods, has the potential to impact on health and medical research across the translational spectrum.

National surveys of health and medical research organisations in Australia highlight that involvement of the community requires improvement across the sector, both at the individual, project and organisational levels [6]. CaRE was originally developed by laboratory-based researchers prior to its expansion to support research from the laboratory to the clinical setting. There is ongoing work internationally to develop frameworks to engage the community in laboratory-based research [18], and there is the existence of many other frameworks to support community engagement across broader health-related research [16, 17]. To this end, intention of the CaRE was not to produce another framework but rather to be a program building local capacity for community engagement in research, including developing partnerships with community members so that they can engage in research as an embedded and meaningful contributor of a research team. The reported impacts of these partnerships on community members is promising for the longevity of the CaRE program as they are consistent with key sources of motivation of the Australian public to engage with health and medical research, such as direct benefit to themselves or their family members, and supporting positive outcomes for others with the similar conditions [22]. The approach supported in CaRE partnerships resembles lessons learnt from previously published community and researcher buddy partnerships (e.g. ensure mutual benefit, build rapport) [23, 24]. The approach to partnerships within CaRE, however, is not limited to career stage or a project (e.g. PhD projects) [23], and the wider program is working with relevant community and charitable organisations to facilitate wider community connection to research through the delivery of public-facing activities. As a program that is still in its infancy, CaRE is continually seeking feedback from community members and has held focus groups to inform new processes where a need has been identified (e.g. development of written and digital guides during onboarding). The dedication of a voluntary CaRE committee to actively listen to community members and follow through with their suggestions and preferences has been crucial to in establishing a program that shares power and fosters authentic engagement.

### Limitations of the findings

It is worth noting that the collection of quantitative and qualitative data of the first three years reported here was designed and conducted solely to define, measure and improve the current program. We did not use any formal research methods to synthesise the free text data obtained from community members and researchers. Whilst content or thematic analysis may have provided more structure or a deeper interpretation of the data, we chose to prioritise transparency by presenting all free text in a tabulated format. With full visibility of the data, we encourage the reader to also draw their own conclusions. The evaluation was not designed to extrapolate the data from CaRE members to the research community at large and the findings are specific to the context of this program, which does limit generalisability. Not all community members and researchers who were enrolled in the CaRE program responded to annual surveys on their satisfaction and experience. Hence, it is conceivable that findings represent people who are the most engaged with the program. Community members who have engaged with the program have been mostly female, but this is similar to national trends of patient and public involvement and engagement activities in the United Kingdom [25, 26].

### Future directions

Since its inception, CaRE has been navigating the inherent challenges of developing and establishing a new community engagement initiative in an academic setting, and these experiences have provided valuable opportunities for reflection and growth. Through ongoing evaluation, CaRE has made incremental steps towards achieving its core goal of building meaningful collaborations with the community but there are areas for future improvement.

One of the key benefits for researchers within CaRE to date has been the ability to connect with the community and gain new insight on the value of involving lived experience in research. Similar to previous literature on supporting community engagement in research on chronic conditions [27], it was evident from the researchers that having a formal community coordinator is integral in supporting the connection with the community members. At the same time, it was evident that not all researchers who have joined the program have been paired with a community member, or those that had been paired found challenges in identifying a “common ground” or a collaboration of mutual benefit. Although based on limited data, feedback from both community members and researchers suggested this occurred when lived experience of the community member was considered far removed from the technical aspects of basic science or that community members had a rarer form of the disease being studied by the researcher. Such barriers to community engagement are not new to laboratory-based research, but community engagement in this space is feasible and can benefit basic science researchers in similar ways to clinical researchers (e.g. mutual learning opportunities, valuing new perspectives) [28]. Nevertheless, there are some important distinctions when engaging the community with laboratory-based research. CaRE can benefit from recent practical strategies published by others including providing case studies to help change mindsets on the link between laboratory science and lived experience as well as allocating more time at the start of partnerships to co-develop a shared vocabulary (e.g. glossary) [29, 30]. Indeed, researchers in the CaRE program did highlight mentoring or support through receiving specific previous examples of successful engagement as ways to enhance their experience.

Ensuring broad reach in the community is a key challenge for programs such as CaRE. CaRE is hosted by a university in a metropolitan area in Victoria, which reflects the presence of most main University campuses in the state of Victoria and wider Australia [31]. Few of the community members in the program resided from regional areas; a clear scope for improvement for CaRE that is relevant to the Australian context and for ensuring health is informed by local need [32]. To support broader reach, CaRE has adopted social media platforms, including X (formerly Twitter), YouTube, Facebook, and LinkedIn, to disseminate information about research advancements and engage with the community. An example was the 2022 Rare Disease Day video series featuring patient stories and expert perspectives on autoimmune encephalitis, Friedreich ataxia, primary immunodeficiency disease, visual snow syndrome, myeloproliferative neoplasms and brain cancer. Additionally, CaRE has hosted webinars and seminars featuring various medical conditions, providing a platform for community members to learn about research and share their experiences. Continuing such activities can help to raise awareness of rare diseases and unmet medical needs in the wider community, but as such work transcends academic and health care settings CaRE will need to adopt accessible and culturally appropriate methods and language to engage with Indigenous Australians and people from culturally and linguistically diverse backgrounds [33].

Community engagement in translational research does not take place in a vacuum. Future activities of CaRE will be influenced by financial support and wider institutional processes. The funding of the CaRE program to date has been limited to a part-time community engagement coordinator position only (0.2 Full-time Equivalent for first 12 months, then 0.4 thereafter). CaRE committee community representatives have not been remunerated. Although some partnerships have benefited from project specific funding obtained by the researchers, no community members in the partnerships have received remuneration directly from the CaRE program. CaRE currently recruits unremunerated community partners into a program that’s designed to help researchers understand, in part, the vital importance of renumerating community members. It is important for CaRE to remain up-to-date with best practice in community engagement by being able to support remuneration of community members for their time and contributions. In fact, some of the researchers within CaRE partnerships have already requested the need for funding. Fair and timely remuneration of community members is an essential aspect of successful engaged research but it is also influenced by complex institutional structures and factors that can be outside of the control of the researchers and community members [34] For example, it is in the experience of the authors that there is a lack of clarity on institutional processes of remunerating community members, which has led to misconceptions that only some remuneration methods are possible (e.g. gift cards vs bank payments), especially when researchers are pursuing remuneration of community members for the first time. Difficulty in navigating institutional processes leading to community members being remunerated by a non-preferred method has been reported previously [35]. The lack of transparency and clarity in payment processes was also recently cited as one of many barriers in the remuneration of community members in research [34]. Billheiemer et al. [34] have posed compelling recommendations to address such barriers, all of which are all broadly relevant to the current state of play within universities in Australia. There are opportunities for CaRE to address some of these areas locally, but the program will also be aided by the work of others. For example, in Australia, there is no need for CaRE to reinvent the wheel and make specific recommendations on rates of pay, this is already provided nationally including centres and alliances based in the state of Victoria (e.g. Australian Clinical Trials Alliance, Monash Partners, Victorian Comprehensive Cancer Centre Alliance). CaRE, however, is preparing a remuneration policy/standard operating procedure to raise awareness of these guidelines, provide specific instructions to navigate the university systems and share standardised payment forms (including choice of methods) and agreement templates to streamline the processing time.

We, lastly, reflect on the cultural shift that is required with community engagement in translational research. The program to date has engaged with researchers who already understood the importance of engaging the community and others who were new to it, benefiting from an increase in enthusiasm and confidence to engage with the community. Nevertheless, one may have expected that the total number of researchers who had joined in the CaRE program over the three years to be larger when the total pool of researchers involved several academic departments and research centres. It would be worthwhile understanding to what extent this is due to researchers already engaged with the community, a lack of awareness of the program, or belief that the community engagement is not relevant to them. Some disciplines are more inclined to engage the community than others [15]. A potential future direction for CaRE is to support the development of condition or department specific community advisory groups or panels, building on individual community member and researcher partnerships. A targeted approach and commitment at a departmental or centre level may offer additional benefits, such as offering a source of sustainable funding, greater buy-in from researchers and increase the likelihood of community engagement across broader activities. Academic behaviours and the success of community engagement practices remain critically dependent on the institutional context [36]. A culture of publish-or-perish continues to dictate academic activities in Australia [37]. Whilst there is desire from universities for researchers to engage the community, reward systems and career progression still prioritise grant and publication metrics [36, 38]. It is evident that many of the contributions of community members in CaRE partnerships were towards funding proposals. It has previously been reported that a driving motivation for researchers to engage the community was the requirement for research funding [11]. While guidance by key Australian funding bodies (e.g. NHMRC, MRFF) encourages community engagement in health and medical research and there have been changes to key assessment criteria, it is not currently mandatory [39]. We contend that such encouragement and changes to assessment criteria have largely been directed at schemes that fund clinical trials and public facing research and will likely do little to influence beliefs regarding community engagement in discovery and laboratory-based research. Another relevant policy in this space, came in May 2023, where it was made mandatory for all Australian public and private Australian health services involved in clinical trials to be independently assessed against the National Clinical Trials Governance Framework [40], which includes specific requirements on partnering with consumers from original design to implementation of clinical trials [41]. Such directives will help to increase the awareness of community engagement in research teams affiliated with health services. CaRE will need to ensure that it continues to build capacity and foster an innate drive for community engagement across all stages of translational research, and as stated elsewhere [30], part of the solution to initiating cultural change in laboratory-based research may be targeting early career researchers and making it an essential part of their training.

## Conclusion

The development of the CaRE program provides an important contribution to the evolving landscape of community engagement in translational research and provides an example for establishment of collaborative community and researcher partnerships across the research translation pipeline. The CaRE program intends to continue supporting community engagement during the planning, delivery and implementation of translational research.

## Electronic supplementary material

Below is the link to the electronic supplementary material.

Supplementary Material 1

## Data Availability

Data sharing is not applicable to this article as no datasets were generated or analysed during the current study.

## Abbreviation

CaRE: Community and Researcher Engagement
