# Supplementary material for: Who CaREs - We do: Development of a community and researcher engagement program in translational medicine
Source: Res Involv Engagem. 2026 Feb 4;12:16. doi: 10.1186/s40900-026-00843-2 (PMC12870369; doi:10.1186/s40900-026-00843-2)
Supplement: Supplementary file 1 — Supplementary Material 1 [file 40900_2026_843_MOESM1_ESM.docx]

# **GRIPP2-SF: Guidance for Reporting Involvement of Patients and the Public– Short Form**

| **Section: topic** | **Item** | **Reported on page** |
| --- | --- | --- |
| 1: Aim | Report the aim of PPI in the study | Background: Page 3 & 4 |
| 2: Methods | Provide a clear description of the methods used for PPI in the study | Methods: Page 4 – 8; Figures 1 - 3 |
| 3: Study results | Outcomes – Report the results of PPI in the study, including both the positive and negative outcomes | Results: Page 9 - 23 Tables 2 - 6 |
| 4: Discussion and conclusions | Outcomes – Comment on the extent to which PPI influenced the study overall. Describe positive and negative effects. | Discussion: Page 24- 29 |
| 5: Reflections/critical perspective | Comment critically on the study, reflecting on the things that went well and those that did not, so others can learn from this experience. | Discussion: Page 24 - 29 |
